# Bioenergetic Status of the Intestinal and Hepatic Cells after Short Term Exposure to Fumonisin B1 and Aflatoxin B1

**DOI:** 10.3390/ijms23136945

**Published:** 2022-06-22

**Authors:** Xiangrong Chen, Mohamed F. Abdallah, Charlotte Grootaert, Andreja Rajkovic

**Affiliations:** 1 Department of Food Technology, Safety and Health, Faculty of Bioscience Engineering, Ghent University, 9000 Ghent, Belgium; xiangrong.chen@ugent.be (X.C.); mohamed.fathi@ugent.be (M.F.A.); charlotte.grootaert@ugent.be (C.G.); 2 Department of Forensic Medicine and Toxicology, Faculty of Veterinary Medicine, Assiut University, Assiut 71515, Egypt

**Keywords:** fumonisin B1, aflatoxin B1, cytotoxicity, mitochondrial toxicity

## Abstract

Fumonisin B1 (FB1) and aflatoxin B1 (AFB1) are frequent contaminants of staple foods such as maize. Oral exposure to these toxins poses health hazards by disrupting cellular signaling. However, little is known regarding the multifaced mitochondrial dysfunction-linked toxicity of FB1 and AFB1. Here, we show that after exposure to FB1 and AFB1, mitochondrial respiration significantly decreased by measuring the oxygen consumption rate (OCR), mitochondrial membrane potential (MMP) and reactive oxygen species (ROS). The current work shows that the integrity of mitochondria (MMP and ROS), that is the central component of cell apoptosis, is disrupted by FB1 and AFB1 in undifferentiated Caco-2 and HepG2 cells as in vitro models for human intestine and liver, respectively. It hypothesizes that FB1 and AFB1 could disrupt the mitochondrial electron transport chain (ETC) to induce mitochondrial dysfunction and break the balance of transferring H^+^ between the mitochondrial inner membrane and mitochondrial matrix, however, the proton leak is not increasing and, as a result, ATP synthesis is blocked. At the sub-toxic exposure of 1.0 µg/mL for 24 h, i.e., a viability of 95% in Caco-2 and HepG2 cells, the mitochondrial respiration was, however, stimulated. This suggests that the treated cells could reserve energy for mitochondrial respiration with the exposure of FB1 and AFB1, which could be a survival advantage.

## 1. Introduction

Mycotoxins are considered among the most frequent toxic contaminants of several types of food and feed all over the world [1]. They are produced by several species of toxigenic fungi under certain environmental conditions, which differ between the fungal species [2]. Among these toxins, fumonisins (FBs) and aflatoxins (AFs) are dominant mycotoxins in maize and maize-based foods [3]. The fungal plant pathogens *Fusarium verticillioides* and *Fusarium proliferatum* are known to produce FBs [4,5,6], while *Aspergillus flavus* and *Aspergillus parasiticus* are among the major producers of AFs [7]. The natural occurrence of these toxins has been recorded at significant concentrations in almost all the countries in the world [1,4]. The FB’s family consists of numerous members, however, (fumonisin B1) FB1 is the most important one as it is the most common and toxic member of FBs [8,9]. Similarly, (aflatoxin B1) AFB1 is the most toxic to humans and prevalent member of the AF family in the agricultural commodities [10,11,12].

Recently, the toxicity of FB1 has aroused widespread public concern [13]. Several studies have shown that FB1 can pose many toxic effects (neurotoxic, hepatotoxic and nephrotoxic) in humans [9,14,15]. The International Agency for Research on Cancer (IARC) classified FB1 as group 2B (possible carcinogenic to humans), since there is no direct established causal association between FB1 exposure and cancer in humans (IARC, 2002). The World Health Organization (WHO) recommends the provisional maximum tolerable daily intake (PMTDI) of 2 μg/kg body weight/day for FBs [16]. As reported, the toxic effect of FB1 is more than 20 μg/mL in Caco-2 and HepG2 cells [17,18]. Several studies showed that the toxic properties of FB1 include profound cytotoxic effects such as increased lipid peroxidation, reactive oxygen species (ROS) production and oxidative DNA damage [9,19,20,21,22]. Intracellular ROS accumulation was induced by FB1 through inhibition of the complex I of the electron transport chain [23]. As a result, it can cause a reduction in the rate of mitochondrial and cellular respirations and an increase in ROS generation in primary rat astrocytes and human neuroblastoma cells [24,25,26,27].

AFB1 has hepatotoxic, immunotoxic, mutagenic, carcinogenic and teratogenic properties [28]. Strong evidence linking AFB1 consumption with hepatocellular carcinoma (HCC) occurrence has led the IARC to classify this mycotoxin as a Group I carcinogen for humans (IARC 2012). The PMTDI of 1 ng AF/Kg body weight (bw)/day may be used as a guidance value in the risk assessment of AF from food [16]. Moreover, most of the conducted research reported that the toxicity of AFB1 in Caco-2 and HepG2 cells occurs at a dose of 3.0 μg/mL [29,30]. AFB1 has been found to induce oxidative radical formation through the cytochrome-activated P450 enzyme system, generating lipid peroxidation and reducing antioxidant enzyme activity [31], resulting in the carcinogenicity. These radicals may induce pathologies by damaging lipids, proteins and DNA [32]. Previously, AFB1 has been reported to induce ROS generation and cause oxidative damage [33]. Induced ROS can cause unstable mitochondrial membrane potential [34]. Indeed, some papers describe that AFB1 can induce cell apoptosis mainly through mitochondria-associated pathways and also impair mitochondrial functions [35,36].

In this study, we aim to further explore the more subtle effects of FB1 and AFB1 on key metabolic processes at subtoxic concentrations to model the impact of low-dose, and hence, more human-relevant, exposure to mycotoxins. To this end, undifferentiated Caco-2 and HepG2 cells as in vitro models for the intestine and liver, respectively, as ‘gatekeepers’ of unwanted substances, were used to evaluate the toxicity of FB1 and AFB1. First line cytotoxicity assays were complemented by studies of the effect of FB1 and AFB1 on the mitochondrial respiration parameters and bioenergetic status of the Caco-2 and HepG2 cells. This study formed the essential basis for future studies of co-exposure and effects of mycotoxins with other contaminants, such as omnipresent microplastics.

## 2. Results

### 2.1. Cytotoxicity of FB1 and AFB1 on Caco-2 and HepG2 upon Different Exposure Times

**Figure 1:**
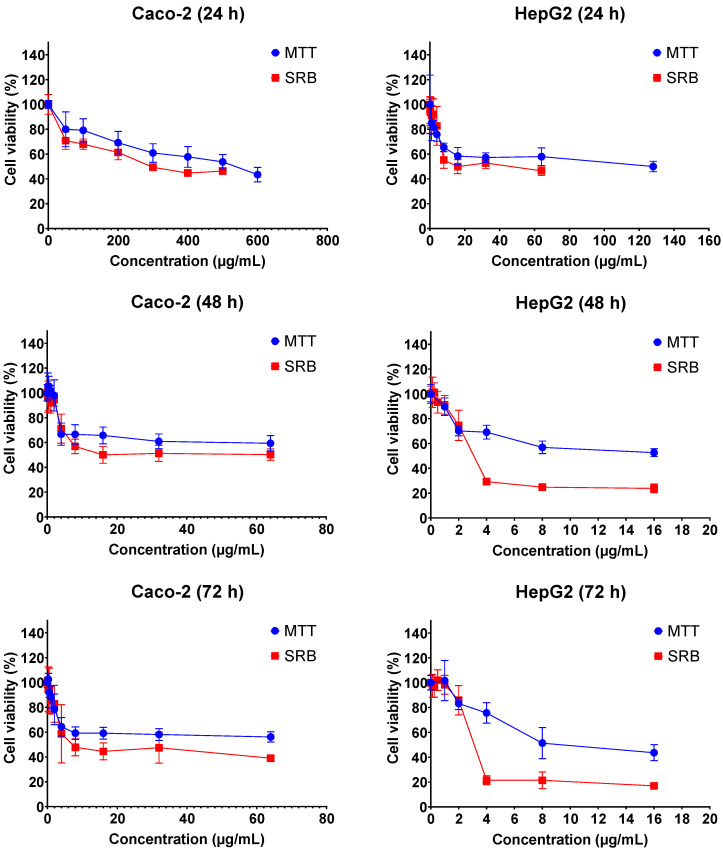
Concentration–response curves for the individual 24 h, 48 h and 72 h treatments with FB1 in Caco-2 and HepG2 cells. Data (three technical replicates and three independent repetitions) are expressed as mean ± SD.

**Figure 2:**
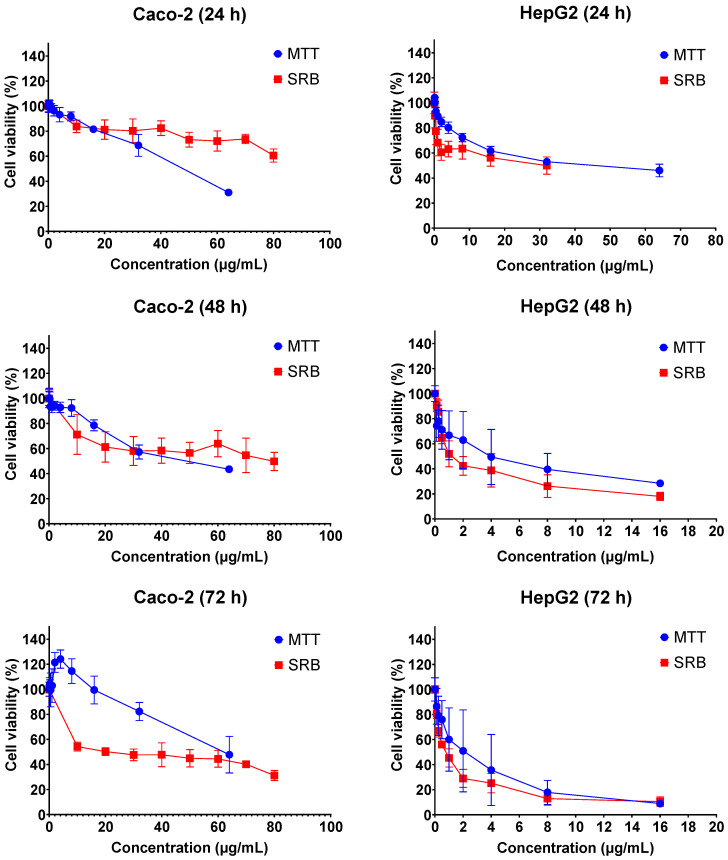
Concentration–response curves for the individual 24 h, 48 h and 72 h treatments with AFB1 in Caco-2 and HepG2 cells. Data (three technical replicates and three independent repetitions) are expressed as mean ± SD.

The cytotoxic effects of FB1 and AFB1 on Caco-2 and HepG2 cells were evaluated by 3-(4,5-dimethylthiazol-2-yl)-2,5-diphenyl-2H-tetrazolium bromide (MTT) and sulforhodamine B (SRB) assays over three different exposure times (24 h, 48 h and 72 h). As shown in Figure 1 and Figure 2, all the tested concentrations have reduced the cell viability in a time- and concentration-dependent manner. In Figure 1, the dose response curve of the SRB assay was still lower than the MTT assay for all treatments of FB1 in Caco-2 and HepG2 cells. A similar phenomenon was also observed when AFB1 treated HepG2 cells for 24 h, 48 h and 72 h (Figure 2).

All IC_50_ values of FB1 and AFB1 on Caco-2 and HepG2 cells were calculated based on the obtained results from Figure 1 and Figure 2 and are shown in Table 1. It was observed that all the IC_50_ values after 24 h of exposure were much higher than those after 48 h and 72 h, except for AFB1 exposure in Caco-2 cells detected by MTT assay. There is a remarkable difference in all the obtained IC_50_ values between 24 h and 48 h treatments, which is not the case between 48 h and 72 h. For this reason, the exposure times (24 h and 48 h) were chosen to study the effects of FB1 and AFB1 on the mitochondrial respiration in Caco-2 and HepG2 cells. To ensure a sufficient number of living cells after 24 h and 48 h of exposure, the maximum concentration of the two toxins was chosen to induce a 65% cell viability in both cells. In addition, another three concentrations were chosen based on cell viabilities of 75%, 85% and 95%.

### 2.2. Different Time Effects of FB1 and AFB1 on Mitochondrial Respiration of Caco-2 and HepG2 Cells

**Figure 3:**
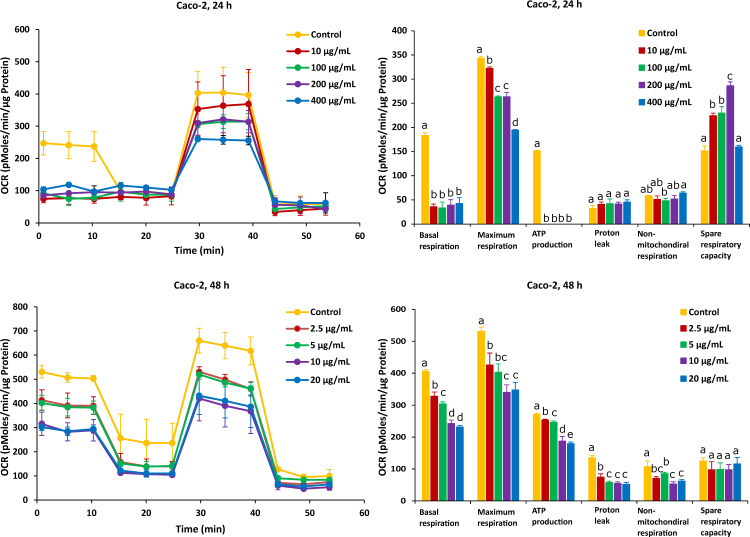
Effect of FB1 on oxygen consumption rate (OCR) (**left**) and different mitochondrial parameters (**right**) in Caco-2 cells after 24 h and 48 h of exposure. Data (at least four technical replicates) are expressed as mean ± standard deviation. Mean values with different letters (a–e) within each mitochondrial parameter indicate significant differences (*p* < 0.05) among different FB1 treatments according to one way ANOVA test followed by Tukey HSD multiple-comparison test as a post hoc analysis.

As shown in Figure 3, after the Caco-2 cells were treated with FB1 for a period of 24 h, the mitochondrial respiration was significantly inhibited. Basal respiration, ATP production and spare respiratory capacity considerably declined regardless of the applied toxic dose of FB1. Moreover, the maximum rate of respiration that the cell can achieve was significantly decreased with increasing FB1 concentration. With all test concentrations of FB1, no difference in both proton leak and non-mitochondrial respiration was observed. For the 48 h of FB1 exposure, it can be seen that the respiratory capacity of the Caco-2 cells has partially recovered compared to the 24 h condition, but also that basal respiration, maximum respiration, ATP production, proton leak and non-mitochondrial respiration significantly decreased depending on FB1 concentrations (Figure 3). The spare respiratory capacity significantly increased at 10, 100 and 200 µg/mL of FB1 in 24 h of exposure, while it became of no significance with all treatments in the case of 48 h of exposure.

**Figure 4:**
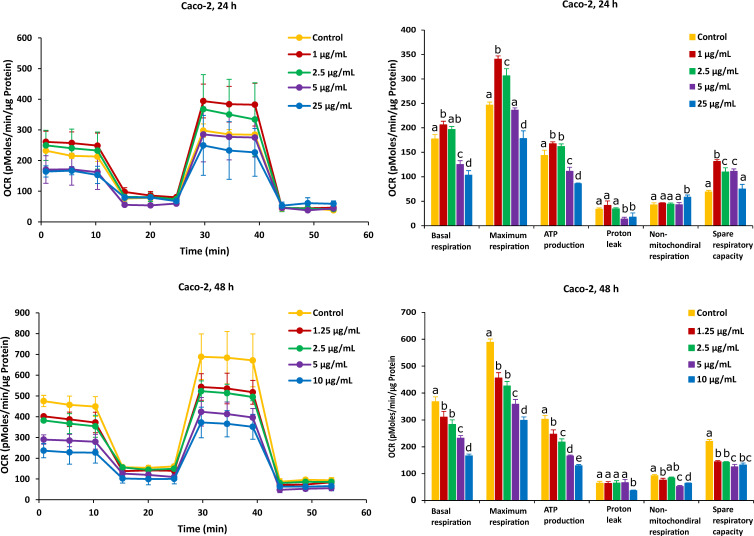
Effect of AFB1 on oxygen consumption rate (OCR) (**left**) and different mitochondrial parameters (**right**) in Caco-2 cells after 24 h and 48 h of exposure. Data (at least four technical replicates) are expressed as mean ± standard deviation. Mean values with different letters (a–e) within each mitochondrial parameter indicate significant differences (*p* < 0.05) among different AFB1 treatments according to one way ANOVA test followed by Tukey HSD multiple-comparison test as a post hoc analysis.

On the other hand, after Caco-2 cells exposure to AFB1 for 24 h with 1 µg/mL and 2.5 µg/mL (the corresponding viabilities are 95% and 85%), mitochondrial respiration was not inhibited, and AFB1 stimulated Caco-2 cells to produce more energetic values, which were observed in all the six mitochondrial parameters (Figure 4). However, after the Caco-2 cells were treated by AFB1 for 48 h with 1.25 µg/mL and 2.5 µg/mL (with corresponding viabilities of 95% and 85%, respectively), all the mitochondrial parameters were significantly decreased. With all the applied concentrations taken together, except proton leak and non-mitochondrial respiration, the mitochondrial parameters were significantly decreased in a concentration dependent manner.

**Figure 5:**
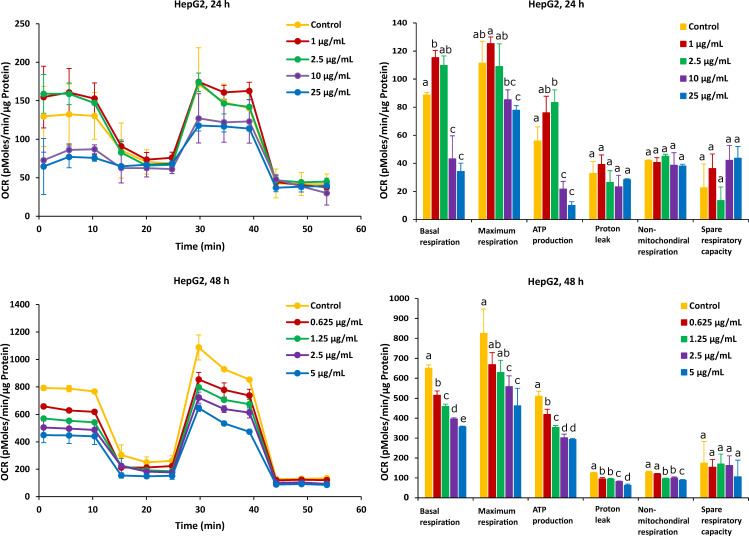
Effect of FB1 on oxygen consumption rate (OCR) (**left**) and different mitochondrial parameters (**right**) in HepG2 cells after 24 h and 48 h of exposure. Data (at least four technical replicates) are expressed as mean ± standard deviation. Mean values with different letters (a–e) within each mitochondrial parameter indicate significant differences (*p* < 0.05) between different FB1 treatments according to one way ANOVA test followed by Tukey (HSD) multiple-comparison test as a post hoc analysis.

**Figure 6:**
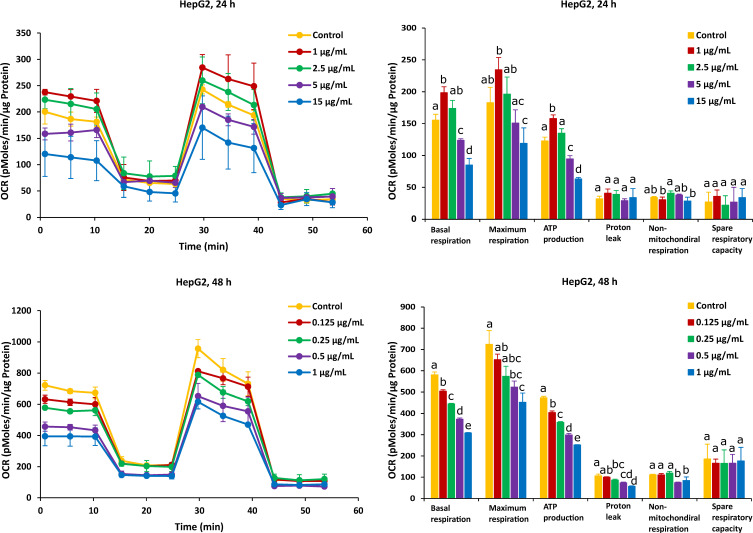
Effect of AFB1 on oxygen consumption rate (OCR) (**left**) and different mitochondrial parameters (**right**) in HepG2 cells after 24 h and 48 h of exposure. Data (at least four technical replicates) are expressed as mean ± standard deviation. Mean values with different letters (a–e) within each mitochondrial parameter indicate significant differences (*p* < 0.05) between different AFB1 treatments according to one way ANOVA test followed by Tukey HSD multiple-comparison test as a post hoc analysis.

For HepG2 cells, the effects on mitochondrial respiration after exposure to FB1 and AFB1 for 24 h and 48 h are shown in Figure 5 and Figure 6, respectively. After 24 h of exposure to any applied dose of FB1 (1, 2.5, 10 and 25 µg/mL) or AFB1 (1, 2.5, 5 and 15 µg/mL), there was a difference in proton leak, non-mitochondrial respiration and spare respiratory capacity. Exposure to FB1 (10 and 25 µg/mL) and AFB1 (5 and 15 µg/mL) results in a significant decrease in basal respiration, maximum respiration and ATP production. A lower concentration of 2.5 µg/mL of either FB1 or AFB1 did not have a significant effect over 24 h, while upon exposure to either FB1 or AFB1 at 1 µg/mL, there was a significant increase in the three mitochondrial parameters (basal respiration, maximal respiration and ATP production). With more prolonged time of exposure (48 h) to FB1 and AFB1, all the mitochondrial parameters were significantly decreased in a concentration dependent manner except spare respiratory capacity. Interestingly, upon FB1 exposure of 48 h, basal respiration was not recovered under the exposure of 0.625 µg/mL (95% viability) compared to the 24 h exposure for 1 µg/mL (95% viability), thereby pointing to an adaptation process to the FB1 stress in these conditions, as was also seen for the Caco-2 cells.

### 2.3. ROS Production and MMP

**Figure 7:**
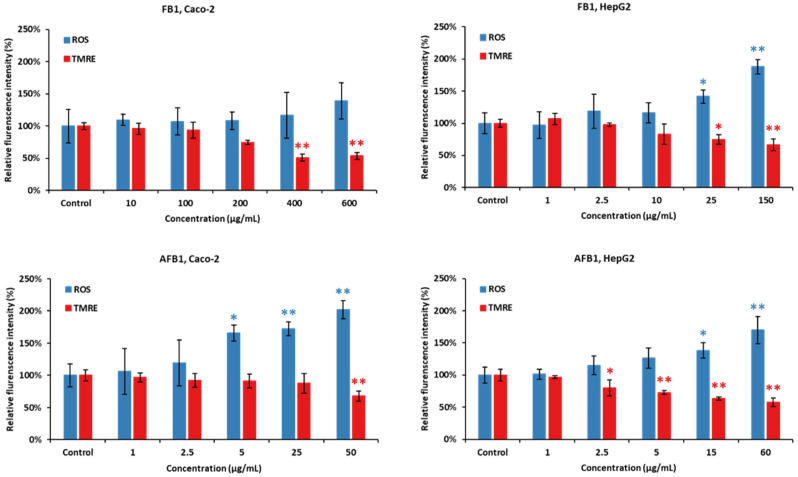
Effect of FB1 and AFB1 on reactive oxygen species (ROS) production and mitochondrial membrane permeability (MMP) in Caco-2 and HepG2 cells after 24 h exposure. Data (at least three technical replicates) are expressed as mean ± standard deviation. Statistic difference at equivalent toxicity is labeled by **: *p* < 0.01; *: *p* < 0.05 versus control by one-way ANOVA with Dunnett’s post hoc test.

As shown in Figure 7, Caco-2 and HepG2 cells were treated with five concentrations of FB1 and AFB1 (the corresponding viability are 95%, 85%, 75%, 65% and 50%) for a period of 24 h, respectively. ROS generation and MMP disruption in the FB1/AFB1-treated cells resulted in a significant and concentration-dependent increase (*p* < 0.05 or 0.01), but no significant increase in ROS generation was induced by FB1 in Caco-2 cells. ROS generation was significantly induced by FB1 (400 µg/mL) and AFB1 (25 µg/mL) in Caco-2 cells. For HepG2 cells, there is a significant decrease after the exposure to FB1 (25 µg/mL) and AFB1 (15 µg/mL), respectively. Similarly, MMP is remarkably disrupted by both mycotoxins, except Caco-2 cells exposure to AFB1 with 25 µg/mL.

## 3. Discussion

Human cell lines are excellent in vitro models for high-throughput toxicity screening. Caco-2 cells feature many characteristics of the intestinal epithelial cells, used as an alternative model to study epithelial-cell invasion and most commonly used to assess toxicity [37,38]. The hepatoma cell line, HepG2, morphologically resembles hepatocytes in addition to its ability to retain many of the enzymes involved in xenobiotic metabolism [39]. Therefore, both cell lines were chosen to investigate the effect of FB1 and AFB1 on the bioenergetic status of the intestine and liver. Our study shows that toxins impact cell bioenergetics and their ability to cope with extra stress and energy demands, and that effect is toxin and cell type specific.

Under our tested conditions, the obtained results clearly demonstrate that FB1 and AFB1 have powerful cytotoxic properties towards Caco-2 and HepG2 cells. We found that the results of the SRB assay were lower than the MTT assay after FB1 and AFB1 exposure to both cells. The MTT, measuring metabolically active cells, and SRB assay, measuring the protein content of both live and dead adhered cells, are combined to assess whether MTT decreases are due to the presence of less cells (anti-proliferative) or to the inhibition of the mitochondrial respiration. In some cases, the MTT values are higher than the SRB values, which may be due to protein losses or over reactivity. It is indeed reported in the literature that there is a delay time between MTT and SRB, resulting in the SRB assay having a 20%–50% lower IC50 value than the MTT assay in short-term cultures [40]. In our study, higher concentrations of FB1 are required to kill half of the cells within 24 h of exposure, while much lower concentrations, in case of longer time of exposure (i.e., 48 h and 72 h), can have the same toxic effect as in 24 h (Table 1). This is in accordance with previous results reported by Wentzel et al., who showed that there is no drastically negative impact on the Caco-2 or HepG2 viability after short-term (24 h) exposure to FB1 at concentrations between 32.1 and 10.7 µg/mL [41]. Furthermore, Cetin and Bullerman have shown that the IC_50_ values for FB1 in Caco2 and HepG2 are more than 100 µg/mL for the exposure times of 48 h and 72 h, however, they did not mention the exact concentrations applied in the MTT assay [42]. Du et al. have shown that the IC_50_ value is 343.9 µg/mL after FB1 exposure in HepG2 cells for 24 h by SRB assay [43], which is similar to our results (Table 1).

For AFB1, previous works reported the IC_50_ values in Caco-2 cells (20.4 µg/mL, 19.7 µg/mL and 19.2 µg/mL for the exposure times of 24 h, 48 h and 72 h, respectively) [30,44], which are lower than our results (Table 1). There was no difference in the IC_50_ values in the three exposure times, which is also the same observation as in our MTT assay (48.0 µg/mL, 52.3 µg/mL and 59.3 µg/mL for the exposure times of 24 h, 48 h and 72 h, respectively). However, in SRB assay, all the AFB1-tested concentrations have reduced the cell viability in a time-dependent manner. It could be that SRB measures protein content of both live and dead cells, whereas MTT only measures live cells. This is the reason why the adhered cells are mostly dead, but are not detached yet. It is worth mentioning that the cell density (6000 cells/well) in the previous works was lower than the cell density used in our experimental setup (20,000 cells/well). The MTT-based IC_50_ value for HepG2 viability after 24 h of exposure was reported before to be 31.25 µg/mL [45], which is half the concentration in our experiment. Furthermore, Liu et al. and Du et al. indicated that the IC_50_ values after 24 h of exposure in HepG2 cells were 6.8 µg/mL and 3.8 µg/mL using SRB assay, respectively, which are much lower than the IC_50_ values in our work [43,46]. Similarly, in MTT, in their studies, the cell density (6000 cells/well and 10,000 cells/well, respectively) was also lower than that used in our experiment (20,000 cells/well). Overall, we may conclude that the seeding density of the cells is an important determinant in the determination of IC_50_ values, which may affect the toxin load per cell and create different ratios of cell populations composed by healthy, damaged and dead cells. Especially, the population of the non-lethally damaged cells may induce a wide variety of cell defense, repair and even apoptosis mechanisms in an attempt to limit tissue damage as much as possible. Amongst these defense systems, bioenergetic shifts are commonly described [47].

**Figure 8:**
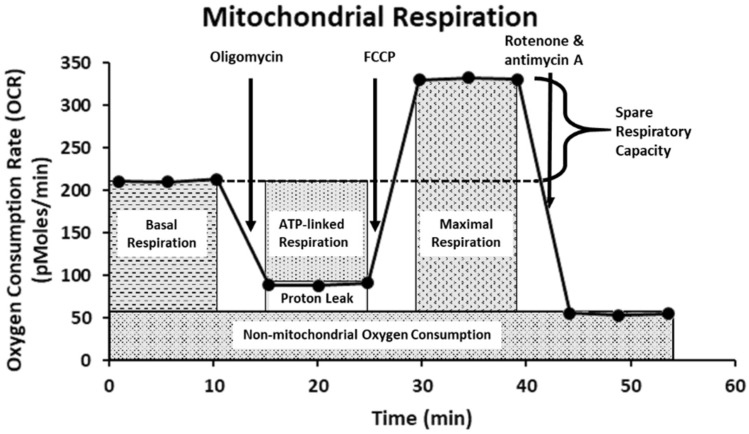
The Agilent Seahorse XF Cell Mito Stress Test profile showing the key parameters of mitochondrial function. This figure is adapted from the Agilent Technologies website.

Mitochondria plays integral roles in energy production, calcium ions (Ca^2+^) and redox homeostasis, as well as regulation of apoptosis [48]. Moreover, emerging evidence also shows that mitochondria are important molecular targets for FB1 and AFB1 [19,24,49]. To investigate the possible toxic effect of FB1 and AFB1 on mitochondrial respiration, we have used the state-of-the-art Agilent Seahorse XF24 Analyzer “Extra-cellular Flux Analysis” to determine the real-time kinetic response of Caco-2 and HepG2 cells. This was indicated through evaluating six fundamental parameters of mitochondrial function: basal respiration, maximal respiration, ATP production, proton leak, non-mitochondrial respiration and spare respiratory capacity. The basal respiration after sequential injection of oligomycin, carbonyl-cyanide-4-(trifluoromethoxy) phenylhydrazine (FCCP) and rotenone + antimycin A is depicted in Figure 8. The Agilent Seahorse XF Cell Mito Stress Test kit is based on the electron transport chain (ETC), which indicates the target of action for all the modulators. Oligomycin inhibits ATP synthase (complex V) and impacts or decreases electron flow through the ETC, resulting in a reduction in mitochondrial respiration or OCR. This decrease in the OCR is linked to the cellular ATP production. FCCP is an uncoupling agent that collapses the proton gradient and disrupts the mitochondrial membrane potential. As a result, electron flow through the ETC is uninhibited, and oxygen consumption by complex IV reaches the maximum. The FCCP-stimulated OCR can then be used to calculate spare respiratory capacity, defined as the difference between maximal respiration and basal respiration. As a consequence, OCR expressed in pMoles/min enables drawing conclusions about the ability to synthesize ATP and about mitochondrial function, even better than the measurements of intermediates (such as ATP or nicotinamide adenine dinucleotide NADH) and potentials [50,51].

In our study, the two exposure times (24 h and 48 h) for FB1 and AFB1 in both Caco-2 and HepG2 cells were considered to investigate the effects of the two toxins on the mitochondrial respiration as the differences in the IC_50_ values were significant, which was not the case for 48 h and 72 h of treatment. Decleer et al. found that the mitochondrial respiration could be barely observed with the Seahorse XF Cell Mito Stress Test when they used the same toxic doses of cereulide toxin, which corresponds to the IC_50_ values from the MTT and SRB assays [52]. Therefore, we applied lower toxic doses in our experiment to give a higher rate of cell viability, and these doses correspond to 65%, 75%, 85% and 95% of cell viability.

The effect of FB1 on mitochondrial respiration was investigated upon 24 h and 48 h treatment of Caco-2 and HepG2 cells by assessing six fundamental parameters of mitochondrial function: basal respiration, maximal respiration, ATP production, proton leak, non-mitochondrial respiration and spare respiratory capacity. In HepG2 cells, the basal respiration significantly increased exposure to the lower concentration (1 µg/mL) over 24 h, while this did not happen in the case of other treatments. This may indicate that under the lower stress of FB1, HepG2 cells could be stimulated to improve the energetic demand in 24 h. A possible explanation for this observation is that HepG2 cells produce more ATP in an attempt to overcome the stress to achieve self-resistance. Loiseau et al. have also stated that basal respiration and FCCP-uncoupled respiration increased significantly in HepG2 treated cells with the low concentration of chloroethyl nitrosourea, suggesting that the treated cells used up a part of their respiratory ‘reserve’ that could be a survival advantage [53]. This is in agreement with the fact that the lower dose of FB1 could affect HepG2 cells to improve the energetic demand to finish the reserve during the mitochondrial respiration. In addition, when cells are exposed to higher doses of FB1, basal respiration was still inhibited in Caco-2 and HepG2 cells. Domijan and Abramov stated that FB1 significantly reduced basal oxygen consumption and led to a decrease in the rate of mitochondrial and cellular respiration [24]. It is possible that FB1 suppresses mitochondrial ETC complexes and breaks the balance of H^+^ transfer between the mitochondrial inner membrane and mitochondrial matrix, which blocks mitochondrial ATP synthesis. Maximal respiration and ATP production are the sensitive parameters for determination of the mitochondrial dysfunction [51]. In our results, there is a significant decrease in all treatments’ exposure to the highest concentration of FB1 in Caco-2 and HepG2 cells, which is consistent to the parameter change in ATP production. It is shown that under the exposure to FB1, the energy demand of both cells is increased due to more ATP required to maintain cellular functions. Therefore, the decrease in maximal respiration and ATP production illustrated that FB1 could cause mitochondrial dysfunction for both cell types. The impact of FB1 on mitochondrial ATP synthase that also translocated protons across the inner membrane has been best observed after the addition of oligomycin. The presence of oligomycin during the estimation of maximal respiration is important to prevent the reverse activity of ATP synthase with rapid intracellular ATP depletion, which may lead to cellular metabolic dysfunction and death [51]. The proton leak was unaffected in both cells over 24 h, and it hypothesizes that FB1 does not cause mitochondrial damage with the chosen FB1 concentrations in our experiment. However, all the applied FB1 concentrations in both Caco-2 and HepG2 cells for 48 h significantly lowered the proton leak in comparison with the untreated control (Figure 3 and Figure 5). During the mitochondrial respiration, the protonmotive force generated during substrate oxidation is not used exclusively to drive ATP synthesis. Some protons “leak” back across the mitochondrial inner membrane, constantly consuming the membrane potential and stimulating activity of the respiratory chain to maintain it, and a high leak may indicate mitochondrial injury [54]. Our results suggest that mitochondrial dysfunctions in FB1 pathogenesis are caused not by proton leak, but maybe by respiratory chain defects, because upon a more prolonged time of FB1 exposure (48 h), the mitochondria failed to regulate ATP production. There was no significant decrease in non-mitochondrial respiration and spare respiratory capacity upon FB1 treatment in both cell types. Non-mitochondrial respiration means that oxygen consumption persists due to a subset of cellular enzymes that continue to consume oxygen after the addition of rotenone and antimycin A. In our study, non-mitochondrial respiration did not change (Figure 3 and Figure 5), which illustrates that our results are an accurate measure of mitochondrial respiration based on the same level of OCR. Spare respiratory capacity was impaired indicating an affected ability of the exposed cells to cope with a sudden increased need for ATP, which is given by the difference between maximal and basal cellular OCR. A cell with a larger spare respiratory capacity can produce more ATP and overcome more stress, including oxidative stress, which indicates that this is estimative of the cell’s ability to cope with large increases in ATP turnover. For Caco-2 cells, FB1 could increase the spare respiratory capacity after 24 h of exposure when Caco-2 cells’ viability was 95%, 85% and 75%. It is hypothesized that the exposure to FB1 over 24 h possibly exerts a positive effect on the ability of Caco-2 cells to cope with the stress in 24 h, while, with a more prolonged time of exposure (48 h) to FB1 or the highest dose of FB1 (400 µg/mL) after 24 h of exposure, cells could lose the ability to cope with a sudden increased need for ATP. For HepG2 cells, the short-term exposure to FB1 possibly exerts negative effects on the ability of cells to cope with stresses (including oxidative stress) in HepG2 cells within 48 h, but it is not obviously observed according to that spare respiratory capacity. It was reported that in HepG2 cells, FB1 upregulated the genes responsive to oxidative stress along with an elevated antioxidant response being mounted [19]. A previous study also stated that spare respiratory capacity levels are critically regulated by cellular signals coming from inside and outside mitochondria, and numerous complimentary signals converge to regulate spare respiratory capacity levels [55]. Those that modify the efficiency of the mitochondrial substrates are involved in short-term mitochondrial plasticity, whereas variations in mitochondrial biogenesis are considered long-term regulators.

The effect of AFB1 on mitochondrial respiration was also exposed for 24 h and 48 h in Caco-2 and HepG2 cells and was indicated through evaluating six fundamental parameters of mitochondrial function. Surprisingly, a low dose of AFB1 (2.5 µg/mL and 1 µg/mL) stimulated the OCR to increase in both cells over 24 h of exposure, which is observed in basal respiration, maximum respiration and ATP production (Figure 4 and Figure 6), while these parameters were significantly inhibited at the same dose in the case of 48 h of exposure. It means that the presence of lower concentrations of AFB1 could stimulate cells to improve the energetic demand in 24 h, while after 48 h exposure, the baseline conditions and basal oxygen consumption were inhibited, resulting in metabolic dysfunction. The significant decrease in maximum respiration and ATP production exposure to AFB1 in both cells over 24 h and 48 h means that ATP synthase or complex IV or V could be inhibited, which may lead to cellular metabolic dysfunction and death. It is hypothesized that AFB1 could negatively affect the complex IV or complex V, and the balance of transferring H^+^ between the mitochondrial inner membrane and mitochondrial matrix was broken. As a consequence, because of the presence of AFB1, cells cannot provide enough H^+^ to synthesize ATP resulting in mitochondrial dysfunction. Previous works also reported that AFB1 decreased the activities of mitochondrial ETC complexes IV and V to disrupt mitochondrial function and ATP production in the liver [56,57,58]. These pieces of evidence are similar to our results, demonstrating that AFB1 could suppress mitochondrial ETC complexes IV or V and block cells’ energy metabolism in the mitochondrial respiration, thus causing mitochondrial dysfunction. The proton leak significantly decreased in the highest concentration of all the applied AFB1 in Caco-2 cells and HepG2 cells over 48 h (Figure 4 and Figure 6), while there is no increase in the proton leak with all treatments. It demonstrates that AFB1 could induce ROS in both cells [59], but not by inducing the proton leak. The high water solubility of AFB1 (range of 11–33 ppm) is likely the reason, since high water solubility makes it different to diffuse into the cell membranes [60]. Sterigmatocystin (ST), and AFB1-structurally similar mycotoxin with a bisdihydrofuran moiety, has similar toxicity to AFB1. ST, with the low water solubility, is more toxic than AFB1 to HepG2 cells. Therefore, it is likely the reason, since low water solubility makes it easy to diffuse into the cells, and the same logic holds for every biological membrane, including that of mitochondria [46]. However, the decrease in proton leak proves that the long-term exposure of AFB1 could lead to the functional decline in cell functionality [61]. The mitochondrial mechanism is important to regulate the mitochondria to produce ATP production, so in Caco-2 cells, ATP decreased much more than in HepG2 cells. Similar to the effect of FB1, there is no significant difference in non-mitochondrial respiration with the exposure to AFB1 over 24 h and 48 h in both cells, except at the highest dose (Figure 4 and Figure 6). That means our results can obtain an accurate measure of mitochondrial respiration based on the same level of OCR. HepG2 cells exposed to AFB1 over 24 h or 48 h showed no significant difference in the spare respiratory capacity, whereas the latter is significantly decreased in Caco-2 cells after 48 h, thereby indicating that the spare respiratory capacity may depend on the cell type. It has been observed that hepatocytes only use approximately 30% of their maximal respiratory capacity to maintain basal respiration [62]. It was observed that there is no significant difference in Caco-2 and HepG2 cells after exposure to AFB1 over 24 h and 48 h. This means that cells have complex mechanisms capable of controlling mitochondrial quality and quantity, such as mitochondrial homeostasis. Marchetti et al. also stated that mitochondrial homeostasis is ensured through coordinated processes, including mitochondrial biogenesis, and these mitochondrial quality control mechanisms regulate spare respiratory capacity levels and ATP transfer [55]. However, AFB1 can still let both Caco-2 and HepG2 cells lose their ability to resist the stress (oxidative stress), and the mitochondrial respiration is disrupted with longer exposure time, as oxidative stress is one of the mechanisms of AFB1-induced cytotoxicity [63]. However, spare respiratory capacity levels are strictly dependent on the cellular context and may not always be observed, or low spare respiratory capacity levels do not always mean mitochondrial dysfunction [55]. Previous studies have also stated that continuous exposure to AFB1 might bring a sustained disturbance in the mitochondrial oxygen consumption that is capable of imparting damage to the mitochondrial membrane, resulting in altered respiratory capacity [57]. It is interesting to highlight the mitochondrial toxicity of AFB1 in case of chronic exposure. It is known that when cells are subjected to stress, energy demand increases when more ATP is required to maintain cellular functions. In addition, AFB1 could still cause mitochondria dysfunction based on the negative effect of mitochondrial oxygen consumption and all mitochondrial parameters.

The mitochondrial membrane potential (MMP) generated by proton pumps (complex I, III and IV) is an essential component in the process of energy storage during oxidative phosphorylation [64]. Reactive oxygen species (ROS) encompass several primary reactive species including superoxide anion (O_2_^−^), hydroxyl radical (OH), hydrogen peroxide (H_2_O_2_) and singlet oxygen (^1^O_2_) [65]. Incomplete electron transfer through ETC complexes I and II results in O_2_^−^ production in the mitochondrial matrix, while electron leak at complex III generates O_2_^−^ in both matrix and intermembrane space [66]. Our results show that FB1 and AFB1 could significantly disrupt the MMP and induce ROS production when FB1 was 400 µg/mL and 25 µg/mL and AFB1 was 25 µg/mL and 15 µg/mL in Caco-2 and HepG2 cells at the same time, respectively. Previous studies have demonstrated that the integrity of mitochondria (MMP and ROS) that is the central component of cell apoptosis is disrupted by FB1 and AFB1 [35,46,67], which is consistent with our results. MMP itself is controlled by electron transport and proton leaks [68]. It is hypothesized that the MMP decrease could be mainly induced by FB1 and AFB1 disrupting electron transport because there is no significant difference in proton leak (Figure 3, Figure 4, Figure 5 and Figure 6). With a decrease in MMP by FB1 and AFB1, the mitochondrial permeability transition pore (PTP) opens, solutes (K^+^, Mg^2+^, and Ca^2+^ ions) and water enter, leading to the swelling of the mitochondrial matrix, rupturing of the outer membrane and subsequent leakage of mitochondrial proteins [69]. Moreover, increased ROS production in mitochondria has been reported upon activation of the PTP, despite the requisite mitochondrial uncoupling [70]. It has been suggested that PTP opening (triggered by Ca^2+^) induces a specific conformational change in complex I and complex III, and it may also inhibit the electron pathway inside complex I and complex III [66]. Finally, Ca^2+^ overload by the MMP decrease can lead to inducing the ROS production and inhibiting the ATP synthesis, resulting in mitochondrial dysfunction and cell death. Indeed, our seahorse results show that when Caco-2 and HepG2 cells were exposed to FB1 (400 µg/mL and 25 µg/mL) and AFB1 (25 µg/mL and 15 µg/mL) for 24 h, ATP production was significantly inhibited. It may demonstrate that FB1 and AFB1 could disrupt the ETC to induce mitochondrial dysfunction.

## 4. Materials and Methods

### 4.1. Chemical Reagents

The mycotoxin FB1 (Cas. No. 116355-83-0; 99% purity) was obtained from Sigma (St. Louis, MO, USA), and AFB1 (Cas. ALX-630-093-M005; >98% purity) was provided by Enzo Life Sciences (Belgium). Stock solutions of FB1 and AFB1 were prepared to 10 mg/mL by dissolving them in distillate dimethyl sulfoxide (DMSO), while working solutions for the experiments were prepared in a cell growth medium at different concentrations. Dulbecco’s modified Eagle’s medium (DMEM), GlutaMAX™, a mixture of penicillin/streptomycin antibiotics and non-essential amino acids (NEAA) were all purchased from Thermo Life Technologies (Merelbeke, Belgium). Fetal Bovine Serum (FBS) was supplied from Greiner Bio-One (Vilvoorde, Belgium). Trypsin-EDTA 0.05% was obtained from Thermo Fisher Scientific (Merelbeke, Belgium), and Dulbecco’s Phosphate Buffered Saline (PBS), without Ca^2+^ and Mg^2+^, was obtained from Westburg (Leusden, The Netherlands).

### 4.2. Cell Culture Materials

Caco-2 (human colorectal adenocarcinoma) and HepG2 (human hepatocellular carcinoma) cells were obtained from the American Type Culture Collection (ATCC, Manassas, VA, USA) and cultured in a DMEM medium (Gibco™, GlutaMAX™) containing 4.5 g/LD-glucose and pyruvate and externally supplemented with FBS (10%), a mixture of penicillin/streptomycin (1%) and NEAA (1%). Cells were grown in T-75 (75 cm^2^) polystyrene cell culture flasks (Thermo Life Technologies, Merelbeke, Belgium) in a humidified chamber with 5–10% CO_2_ at 37 ℃ and 95% air atmosphere at constant humidity. The growth culture medium was changed every 2–3 days and cell morphology was checked by visual inspection with phase-contrast microscopy (Leica DMIC, Leica Microsystem GMbH, Wetzlar, Germany). When the degree of confluence reaches approximately 80% (every 4–6 days), cells were subcultured to maintain their undifferentiated character and, therefore, the rapid growth of the cells. Consequently, Caco-2 and HepG2 cells were gently rinsed with prewarmed PBS for a few seconds, detached from the flask with 4 mL of prewarmed trypsin–EDTA 0.05% for 2–3 min and seeded in a new T-75 flask (ratio 1:5). The passage number of the cells used in this study was maintained between 12 and 25.

### 4.3. Cell Viability and Protein Content Assays

The tetrazolium salt (MTT) (Life Technologies Corporation, Eugene, OR, USA) assay, which is based on the cellular conversion of 3-(4,5-dimethylthiazol-2-yl)-2,5-diphenyltetrazolium bromide into formazan, was performed to determine the cell viability after the cell exposure to FB1 and AFB1, while the sulforhodamine B (Sigma-Aldrich, Co., St. Louis, MO, USA) colorimetric assay (SRB) was conducted to measure the protein content in the Caco-2 and HepG2 cells [71,72]. Briefly, Caco-2 and HepG2 cells were seeded in 96-well plates at a density of 20,000 per well for 24 h before the treatment at 37 ℃ in a sterilized incubator with a humidified atmosphere of 5% CO_2_ to allow the cells’ adhesion. FB1 and AFB1 were diluted with DMEM at different concentrations (0.125–512 µg/mL), and 200 µL were added. The plates were incubated for 24 h, 48 h or 72 h.

For MTT, 100 µL from the mycotoxin-containing medium as well as the untreated control were removed from each well and 20 µL of 5 mg/mL of MTT solution dissolved in PBS were added to each well. The plate was incubated for 2 h at 37 ℃ to convert MTT to formazan. After the incubation, all the media were carefully aspirated, and formazan crystals were solubilized in 200 µL of DMSO. The plates were gently shaken using a MTS 2/4 digital microtiter shaker (IKA-WERKE, Stuttgart, Germany) for 5 min to achieve complete dissolution of formazan crystals. The absorbance was measured at a wavelength of 570 nm by SpectraMax™ Microplate Reader (Molecular Devices, Berkshire, UK). For SRB, the cells were fixed by adding 50 µL of 50% trichloroacetic acid (BioXtra, ≥99.0%, Sigma-Aldrich) solution and incubated at 4 ℃ for 1 h in the dark. After the incubation, the content was discarded and gently washed 4–5 times with tap water. The plate was air-dried and 50 µL SRB (0.4% in 1% glacial acetic acid) solution was added to stain the cells. The plate was incubated at room temperature for 30 min, washed 4-5 times with 1% glacial acetic acid and then air-dried. In the end, 200 µL of a 10 mM Tris (hydroxymethyl)-aminomethane buffer were added to each well and the plates were gently shaken for 10 min. The absorbance was measured at a wavelength of 490 nm with the SpectraMax™ instrument. The results were presented as the percentage of viability (%) relative to the untreated control in MTT and SRB assays based on the following formula:

$$\mathrm{Percentage}\;\mathrm{of}\;\mathrm{viability}\;\left(\%\right)=\frac{\mathrm{absorbance}\;\mathrm{of}\;\mathrm{treated}\;\mathrm{cells}-\mathrm{absorbance}\;\mathrm{blank}}{\mathrm{absorbance}\;\mathrm{of}\;\mathrm{control}-\mathrm{absorbance}\;\mathrm{blank}}\times 100$$

For each treatment concentration in each assay, at least three wells per treatment were considered per plate, and three plates cultured at different time points considered. At last, each treatment was chosen in the optimal range concentration to calculate the IC_50_.

### 4.4. Seahorse Extra-Cellular Flux Analysis of Mitochondrial Respiration

Mitochondrial respiration in Caco-2 and HepG2 cells was characterized as an indicator of cellular metabolism and fitness, in response to FB1 and AFB1 exposure by extra-cellular flux analysis using Agilent Seahorse XF24 Analyzer (Agilent Seahorse Bioscience, Santa Clara, CA, USA). For this purpose, the Agilent Seahorse XF Cell Mito Stress Test was applied to both Caco-2 and HepG2 cells, and OCR was measured in function of time and respiration modulators added. In short, 24 h before the assay, cells were harvested from the T-75 flasks and seeded into a Seahorse 24-well XF Cell Culture microplate (Agilent Seahorse Bioscience, Santa Clara, CA, USA) in 250 µL of the culture medium at a density of 20,000/well. The cells were incubated in a sterilized incubator with 10% CO_2_ at 37 °C and 95% air atmosphere at constant humidity. In parallel, a Seahorse XF Sensor Cartridge was hydrated one day before running the XF Assay by filling each well of the XF Utility Plate with 1 mL of Seahorse XF Calibrant Solution. The hydrated cartridge was kept in an incubator at 37 °C without CO_2_ for 24 h to remove CO_2_ from the media that may interfere with measurements. On the day of analysis, unbuffered XF Assay Media (Agilent Seahorse Bioscience, Santa Clara, CA, USA) was used for extracellular flux measurements. Therefore, the cells were washed twice with non-buffered DMEM supplemented with 10 mM glucose, 2 mM sodium pyruvate and 2 mM glutamine (adjusted to pH 7.4) and then maintained in 450 µL/well of XF assay media at 37 °C in a non-CO_2_ incubator for 1 h, which is necessary for de-gassing the plate, allowing for CO_2_ diffusion from the cells, medium and plates. The mitochondrial function of the cells was analyzed by sequential injections of modulators (with final concentration in the wells); oligomycin (1 µM) was used to block ATP synthase, FCCP (0.25 µM) was used to make the inner mitochondrial membrane permeable for protons and allow maximum electron flux through the ETC, and a mix of rotenone (0.5 µM) and antimycin A (0.5 µM) was used together to inhibit complexes I and III, respectively. These compounds were suspended in a prewarmed XF Assay Medium and loaded into the designated injection ports of the hydrated sensor cartridge corresponding to the order of injection.

The loaded XF Sensor Cartridge with the XF Utility Plate was placed into the XF24 Analyzer and calibrated. After calibration, the XF Utility Plate with the calibration fluid was replaced with the plate containing cells. Each measurement cycle consisted of 1 min of mixing, 1 min of waiting and 2 min of OCR measurements. First, three basal OCR measurements were performed before the addition of modulators, followed by the sequential addition of oligomycin, FCCP and rotenone/antimycin A. Measurement cycles were performed after each addition of given compounds. Through the use of mitochondrial inhibitors, four mitochondrial respiration parameters were determined: basal respiration, ATP production-linked, maximum respiration and proton leak-linked OCR. Finally, to normalize the obtained data to a commonly shared parameter for a correct comparison, an SRB assay was performed after running the Agilent Seahorse XF24 Analyzer.

### 4.5. Cytotoxicity Endpoint Measurement

Caco-2 and HepG2 cells were seeded at a density of 10^5^ cells/mL in a black 96-well plate. After the seeded cells were incubated with FB1 and AFB1 for 24 h, ROS and MMP were measured to reflect the toxic effect of FB1 and AFB1 on both cells. ROS generation was measured using a fluorescent probe (2′,7′-dichlorodihydrofluorescein diacetate (DCFH-DA)) (Sigma-Aldrich), as described in literature [73]. ROS production was expressed as the fluorescence intensity ratio of the treated samples over the appropriate controls (fold increase over control). To evaluate changes in MMP, after all treatments, cells were continued with 900 nM of the fluorescent probe Tetramethylrhodamine ethyl ester (TMRE) for another 0.5 h at 37 °C. Afterwards, cells were washed and resuspended in PBS with 0.2% two times, and the TMRE fluorescence was measured by micro-plate reader using excitation and emission wavelengths of 549 and 575 nm. Results were expressed as the relative TMRE mean fluorescence intensity (fold) in treated cells with respect to the control (untreated ones).

### 4.6. Data Analysis

Excel^©^ for Microsoft Office 365 (Microsoft Corporation, Redmond, WA, USA) was used to normalize the obtained data, and the SPSS software package (SPSS Statistics 27, USA) was used for the statistical evaluation. Comparisons between the untreated control and different FB1 and AFB1 treatments within each mitochondrial parameter (basal respiration, maximal respiration, ATP production, proton leak, non-mitochondrial respiration, spare respiratory capacity) were performed using a one-way analysis of variance (ANOVA) test followed by Tukey HSD multiple-comparison test as a post hoc analysis to identify the sources of detected significance (*p* < 0.05). Data are presented as mean ± standard deviation (SD).

## 5. Conclusions

The cytotoxic effect of FB1 and AFB1 on Caco-2 and HepG2 cells was evaluated over 24 h, 48 h and 72 h, and they reduced the cell viability in a time- and concentration-dependent manner, which confirms the IC_50_ values for FB1 and AFB1 in different cells and at different time points. Based on this, the toxic impact may be underestimated or go unnoticed by MTT/SRB assay alone. Seahorse XF extracellular flux analysis of mitochondrial respiration is used for the identification of mitochondrial toxicity of FB1 and AFB1. When no significant reduction in cell viability was observed based on MTT and SRB results, the Seahorse XF Technology was able to detect reduced maximal respiration. Based on MTT and SRB analyses, four concentrations of FB1 and AFB1 were chosen based on the cell viabilities of 95%, 85%, 75% and 65% to analyze the mitochondrial respiration. Mitochondrial damage has been proposed as the mechanism underlying the induced hepatotoxicity and intestinal cell damage associated with FB1 and AFB1 intoxication. In vitro studies have shown that both Caco-2 and HepG2 cells are highly susceptible to the toxic effect of FB1 and AFB1 in 24 h and 48 h. This was indicated through evaluating six fundamental parameters of mitochondrial function: basal respiration, maximal respiration, ATP production, proton leak, non-mitochondrial respiration and spare respiratory capacity. It demonstrates that FB1 and AFB1 could suppress mitochondrial ETC complexes and break the balance of transferring H^+^ between the mitochondrial inner membrane and mitochondrial matrix. This could block mitochondria to synthesize ATP for the respiration, thus causing mitochondrial dysfunction. The integrity of mitochondria (MMP and ROS) that is the central component of cell apoptosis is disrupted by FB1 and AFB1 in Caco-2 and HepG2 cells, respectively, which demonstrates that both mycotoxins could disrupt the ETC to induce mitochondrial dysfunction. In addition, our research displays that when the viability of Caco-2 and HepG2 cells is 95% in 24 h, mitochondrial respiration was stimulated to produce more energy. This study shows for the first time that toxins impact cell bioenergetics and their ability to cope with extra stress and energy demands, and that this effect is toxin and cell type specific. We may, therefore, suggest that prolonged exposure to these toxins, even at lower doses, may contribute to chronic intestinal dysfunction at suboptimal health conditions. The obtained data offer an in-depth view of mitochondrial effects of mycotoxins in human intestinal and liver cell models, allowing further investigations of effects of oral co-exposure to microbiological and chemical mixtures at the nexus of environment, food and health, comprising mycotoxins, metals, allergens, antibiotics and microplastics.

## Figures and Tables

**Table 1:** IC_50_ values (± SD) of FB1 and AFB1 in Caco-2 and HepG2 cells after 24 h, 48 h and 72 h of exposure.

Mycotoxin	Cell	Exposure Time	MTT_IC_50_ (µg/mL)	SRB_IC_50_ (µg/mL)
Fumonisin B1	Caco-2	24 h	589.7 ± 23.4	369.2 ± 12.1
		48 h	158.7 ± 38.4	37.9 ± 11.8
		72 h	52.9 ± 13.6	19.0 ± 11.19
	HepG2	24 h	156.2 ± 16.8	33.9 ± 11.2
		48 h	14.9 ± 15.7	3.5 ± 0.81
		72 h	9.1 ± 10.1	3.0 ± 1.1
Aflatoxin B1	Caco-2	24 h	48.0 ± 3.2	119.2 ± 11.0
		48 h	52.3 ± 3.3	58.0 ± 7.7
		72 h	59.3 ± 5.7	22.8 ± 11.7
	HepG2	24 h	63.6 ± 23.3	23.5 ± 14.7
		48 h	3.6 ± 3.9	1.6 ± 0.4
		72 h	2.0 ± 2.2	0.7 ± 0.1

## Data Availability

Not applicable.

## References

[1] Alshannaq A., Yu J.H. (2017). Occurrence, Toxicity, and Analysis of Major Mycotoxins in Food. Int. J. Environ. Res. Public Health.

[2] Moretti A., Logrieco A.F., Susca A. (2017). Mycotoxins: An Underhand Food Problem. Methods Mol. Biol..

[3] Eskola M., Kos G., Elliott C.T., Hajšlová J., Mayar S., Krska R. (2020). Worldwide Contamination of Food-Crops with Mycotoxins: Validity of the Widely Cited ‘FAO Estimate’ of 25%. Crit. Rev. Food Sci. Nutr..

[4] Ji F., He D., Olaniran A.O., Mokoena M.P., Xu J., Shi J. (2019). Occurrence, Toxicity, Production and Detection of Fusarium Mycotoxin: A Review. Food Prod. Process. Nutr..

[5] Rheeder J.P., Marasas W.F.O., Vismer H.F. (2002). Production of Fumonisin Analogs by Fusarium Species. Appl. Environ. Microbiol..

[6] Chen X., Landschoot S., Detavernier C., De Saeger S., Rajkovic A., Audenaert K. (2021). Cross-Talk between Fusarium Verticillioides and Aspergillus Flavus in Vitro and in Planta. Mycotoxin Res..

[7] Amaike S., Keller N.P. (2011). Aspergillus Flavus. Annu. Rev. Phytopathol..

[8] Scott P.M. (2012). Recent Research on Fumonisins: A Review. Food Addit. Contam. Part A Chem. Anal. Control. Expo. Risk Assess..

[9] Stockmann-Juvala H., Savolainen K. (2008). A Review of the Toxic Effects and Mechanisms of Action of Fumonisin B 1. Hum. Exp. Toxicol..

[10] Abrar M., Anjum F.M., Butt M.S., Pasha I., Randhawa M.A., Saeed F., Waqas K. (2013). Aflatoxins: Biosynthesis, Occurrence, Toxicity, and Remedies. Crit. Rev. Food Sci. Nutr..

[11] Ismail A., Gonçalves B.L., de Neeff D.V., Ponzilacqua B., Coppa C.F.S.C., Hintzsche H., Sajid M., Cruz A.G., Corassin C.H., Oliveira C.A.F. (2018). Aflatoxin in Foodstuffs: Occurrence and Recent Advances in Decontamination. Food Res. Int..

[12] Demissie N. (2018). A Review of Aflatoxin: Occurrence, Prevention, and Gaps in Both Food and Feed Safety. Nov. Tech. Nutr. Food Sci..

[13] Lumsangkul C., Chiang H.I., Lo N.W., Fan Y.K., Ju J.C. (2019). Developmental Toxicity of Mycotoxin Fumonisin B 1 in Animal Embryogenesis: An Overview. Toxins.

[14] Voss K.A., Smith G.W., Haschek W.M. (2007). Fumonisins: Toxicokinetics, Mechanism of Action and Toxicity. Anim. Feed Sci. Technol..

[15] Chen J., Wen J., Tang Y., Shi J., Mu G., Yan R., Cai J., Long M. (2021). Research Progress on Fumonisin B1 Contamination and Toxicity: A Review. Molecules.

[16] World Health Organization (2012). Safety Evaluation of Certain Food Additives and Contaminants: Prepared by the Seventy Fourth Meeting of the Joint FAO/WHO Expert Committee on Food Additives (JECFA).

[17] Abdul N.S., Chuturgoon A.A. (2021). Fumonisin B1 Regulates LDL Receptor and ABCA1 Expression in an LXR Dependent Mechanism in Liver (HepG2) Cells. Toxicon.

[18] Kouadio J.H., Mobio T.A., Baudrimont I., Moukha S., Dano S.D., Creppy E.E. (2005). Comparative Study of Cytotoxicity and Oxidative Stress Induced by Deoxynivalenol, Zearalenone or Fumonisin B1 in Human Intestinal Cell Line Caco-2. Toxicology.

[19] Arumugam T., Pillay Y., Ghazi T., Nagiah S., Abdul N.S., Chuturgoon A.A. (2019). Fumonisin B 1 -Induced Oxidative Stress Triggers Nrf2-Mediated Antioxidant Response in Human Hepatocellular Carcinoma (HepG2) Cells. Mycotoxin Res..

[20] Singh M.P., Kang S.C. (2017). Endoplasmic Reticulum Stress-Mediated Autophagy Activation Attenuates Fumonisin B1 Induced Hepatotoxicity in Vitro and in Vivo. Food Chem. Toxicol..

[21] Stockmann-Juvala H., Mikkola J., Naarala J., Loikkanen J., Elovaara E., Savolainen K. (2004). Oxidative Stress Induced by Fumonisin B1 in Continuous Human and Rodent Neural Cell Cultures. Free Radic. Res..

[22] Domijan A.M. (2012). Fumonisin B1: A Neurotoxic Mycotoxin. Arh. Hig. Rada Toksikol..

[23] Marí M., Morales A., Colell A., García-Ruiz C., Fernández-Checa J.C. (2009). Mitochondrial Glutathione, a Key Survival Antioxidant. Antioxid. Redox Signal..

[24] Domijan A.M., Abramov A.Y. (2011). Fumonisin B1 Inhibits Mitochondrial Respiration and Deregulates Calcium Homeostasis—Implication to Mechanism of Cell Toxicity. Int. J. Biochem. Cell Biol..

[25] Mary V.S., Theumer M.G., Arias S.L., Rubinstein H.R. (2012). Reactive Oxygen Species Sources and Biomolecular Oxidative Damage Induced by Aflatoxin B1 and Fumonisin B1 in Rat Spleen Mononuclear Cells. Toxicology.

[26] Antonissen G., Devreese M., De Baere S., Martel A., Van Immerseel F., Croubels S. (2017). Impact of Fusarium Mycotoxins on Hepatic and Intestinal MRNA Expression of Cytochrome P450 Enzymes and Drug Transporters, and on the Pharmacokinetics of Oral Enrofloxacin in Broiler Chickens. Food Chem. Toxicol..

[27] Kim S.H., Singh M.P., Sharma C., Kang S.C. (2018). Fumonisin B1 Actuates Oxidative Stress-Associated Colonic Damage via Apoptosis and Autophagy Activation in Murine Model. J. Biochem. Mol. Toxicol..

[28] Meissonnier G.M., Pinton P., Laffitte J., Cossalter A.M., Gong Y.Y., Wild C.P., Bertin G., Galtier P., Oswald I.P. (2008). Immunotoxicity of Aflatoxin B1: Impairment of the Cell-Mediated Response to Vaccine Antigen and Modulation of Cytokine Expression. Toxicol. Appl. Pharmacol..

[29] Desaulniers D., Cummings-Lorbetskie C., Leingartner K., Xiao G.H., Zhou G., Parfett C. (2021). Effects of Vanadium (Sodium Metavanadate) and Aflatoxin-B1 on Cytochrome P450 Activities, DNA Damage and DNA Methylation in Human Liver Cell Lines. Toxicol. In Vitro.

[30] Zhang J., Zheng N., Liu J., Li F.D., Li S.L., Wang J.Q. (2015). Aflatoxin B1 and Aflatoxin M1 Induced Cytotoxicity and DNA Damage in Differentiated and Undifferentiated Caco-2 Cells. Food Chem. Toxicol..

[31] Erdélyi M., Balogh K., Pelyhe C., Kövesi B., Nakade M., Zándoki E., Mézes M., Kovács B. (2018). Changes in the Regulation and Activity of Glutathione Redox System, and Lipid Peroxidation Processes in Short-Term Aflatoxin B1 Exposure in Liver of Laying Hens. J. Anim. Physiol. Anim. Nutr..

[32] Schieber M., Chandel N.S. (2014). ROS Function in Redox Signaling and Oxidative Stress. Curr. Biol..

[33] Wu J., Gan Z., Zhuo R., Zhang L., Wang T., Zhong X. (2020). Resveratrol Attenuates Aflatoxin B1-Induced Ros Formation and Increase of M6a Rna Methylation. Animals.

[34] Zorov D.B., Juhaszova M., Sollott S.J. (2006). Mitochondrial ROS-Induced ROS Release: An Update and Review. Biochim. Biophys. Acta Bioenerg..

[35] Liu Y., Wang W. (2016). Aflatoxin B1 Impairs Mitochondrial Functions, Activates ROS Generation, Induces Apoptosis and Involves Nrf2 Signal Pathway in Primary Broiler Hepatocytes. Anim. Sci. J..

[36] Ge J., Yu H., Li J., Lian Z., Zhang H., Fang H., Qian L. (2017). Assessment of Aflatoxin B1 Myocardial Toxicity in Rats: Mitochondrial Damage and Cellular Apoptosis in Cardiomyocytes Induced by Aflatoxin B1. J. Int. Med. Res..

[37] Ganan M., Collins M., Rastall R., Hotchkiss A.T., Chau H.K., Carrascosa A.V., Martinez-Rodriguez A.J. (2010). Inhibition by Pectic Oligosaccharides of the Invasion of Undifferentiated and Differentiated Caco-2 Cells by Campylobacter Jejuni. Int. J. Food Microbiol..

[38] Ude V.C., Brown D.M., Viale L., Kanase N., Stone V., Johnston H.J. (2017). Impact of Copper Oxide Nanomaterials on Differentiated and Undifferentiated Caco-2 Intestinal Epithelial Cells; Assessment of Cytotoxicity, Barrier Integrity, Cytokine Production and Nanomaterial Penetration. Part. Fibre Toxicol..

[39] Lenaerts K., Bouwman F.K., Lamers W.H., Renes J., Mariman E.C. (2007). Comparative Proteomic Analysis of Cell Lines and Scrapings of the Human Intestinal Epithelium. BMC Genom..

[40] Haselsberger K., Peterson D.C., Thomas D., Darling J.L. (1996). Assay of Anticancer Drugs in Tissue Culture: Comparison of a Tetrazolium-Based Assay and a Protein Binding Dye Assay in Short-Term Cultures Derived from Human Malignant Glioma. Anticancer Drugs.

[41] Wentzel J.F., Lombard M.J., Du Plessis L.H., Zandberg L. (2017). Evaluation of the Cytotoxic Properties, Gene Expression Profiles and Secondary Signalling Responses of Cultured Cells Exposed to Fumonisin B1, Deoxynivalenol and Zearalenone Mycotoxins. Arch. Toxicol..

[42] Cetin Y., Bullerman L.B. (2005). Cytotoxicity of Fusarium Mycotoxins to Mammalian Cell Cultures as Determined by the MTT Bioassay. Food Chem. Toxicol..

[43] Du M., Liu Y., Zhang G. (2017). Interaction of Aflatoxin B1 and Fumonisin B1 in HepG2 Cell Apoptosis. Food Biosci..

[44] Ji J., Wang Q., Wu H., Xia S., Guo H., Blaženović I., Zhang Y., Sun X. (2019). Insights into Cellular Metabolic Pathways of the Combined Toxicity Responses of Caco-2 Cells Exposed to Deoxynivalenol, Zearalenone and Aflatoxin B1. Food Chem. Toxicol..

[45] Corcuera L.A., Arbillaga L., Vettorazzi A., Azqueta A., López de Cerain A. (2011). Ochratoxin A Reduces Aflatoxin B1 Induced DNA Damage Detected by the Comet Assay in Hep G2 Cells. Food Chem. Toxicol..

[46] Liu Y., Du M., Zhang G. (2014). Proapoptotic Activity of Aflatoxin B1 and Sterigmatocystin in HepG2 Cells. Toxicol. Rep..

[47] Fulda S., Gorman A.M., Hori O., Samali A. (2010). Cellular Stress Responses: Cell Survival and Cell Death. Int. J. Cell Biol..

[48] Vakifahmetoglu-Norberg H., Ouchida A.T., Norberg E. (2017). The Role of Mitochondria in Metabolism and Cell Death. Biochem. Biophys. Res. Commun..

[49] Bionda C., Portoukalian J., Schmitt D., Rodriguez-Lafrasse C., Ardail D. (2004). Subcellular Compartmentalization of Ceramide Metabolism: MAM (Mitochondria-Associated Membrane) and/or Mitochondria?. Biochem. J..

[50] Dmitriev R.I., Papkovsky D.B. (2012). Optical Probes and Techniques for O_2_ Measurement in Live Cells and Tissue. Cell. Mol. Life Sci..

[51] Brand M.D., Nicholls D.G. (2011). Assessing Mitochondrial Dysfunction in Cells. Biochem. J..

[52] Decleer M., Jovanovic J., Vakula A., Udovicki B., Agoua R.-S.S.E.K., Madder A., De Saeger S., Rajkovic A. (2018). Oxygen Consumption Rate Analysis of Mitochondrial Dysfunction Caused by Bacillus Cereus Cereulide in Caco-2 and HepG2 Cells. Toxins.

[53] Loiseau D., Morvan D., Chevrollier A., Demidem A., Douay O., Reynier P., Stepien G. (2009). Mitochondrial Bioenergetic Background Confers a Survival Advantage to HepG2 Cells in Response to Chemotherapy. Mol. Carcinog..

[54] Jastroch M., Divakaruni A.S., Mookerjee S., Treberg J.R., Brand M.D. (2010). Mitochondrial Proton and Electron Leaks. Essays Biochem..

[55] Marchetti P., Fovez Q., Germain N., Khamari R., Kluza J. (2020). Mitochondrial Spare Respiratory Capacity: Mechanisms, Regulation, and Significance in Non-Transformed and Cancer Cells. FASEB J..

[56] Wan X.L., Li N., Chen Y.J., Chen X.S., Yang Z., Xu L., Yang H.M., Wang Z.Y. (2021). Protective Effects of Lycopene on Mitochondrial Oxidative Injury and Dysfunction in the Liver of Aflatoxin B1-Exposed Broilers. Poult. Sci..

[57] Shi D., Guo S., Liao S., Su R., Guo M., Liu N., Li P., Tang Z. (2012). Protection of Selenium on Hepatic Mitochondrial Respiratory Control Ratio and Respiratory Chain Complex Activities in Ducklings Intoxicated with Aflatoxin B1. Biol. Trace Elem. Res..

[58] Xu F., Li Y., Cao Z., Zhang J., Huang W. (2021). AFB1-Induced Mice Liver Injury Involves Mitochondrial Dysfunction Mediated by Mitochondrial Biogenesis Inhibition. Ecotoxicol. Environ. Saf..

[59] Guerra M.C., Galvano F., Bonsi L., Speroni E., Costa S., Renzulli C., Cervellati R. (2005). Cyanidin-3- O -β-Glucopyranoside, a Natural Free-Radical Scavenger against Aflatoxin B1- and Ochratoxin A-Induced Cell Damage in a Human Hepatoma Cell Line (Hep G2) and a Human Colonic Adenocarcinoma Cell Line (CaCo-2). Br. J. Nutr..

[60] Wang M., Maki C.R., Deng Y., Tian Y., Phillips T.D. (2017). Development of High Capacity Enterosorbents for Aflatoxin B1 and Other Hazardous Chemicals. Chem. Res. Toxicol..

[61] Ramsey J.J., Hagopian K., Kenny T.M., Koomson E.K., Bevilacqua L., Weindruch R., Harper M.E. (2004). Proton Leak and Hydrogen Peroxide Production in Liver Mitochondria from Energy-Restricted Rats. Am. J. Physiol. Endocrinol. Metab..

[62] Hill B.G., Benavides G.A., Lancaster J.J.R., Ballinger S., Dell’Italia L., Zhang J., Darley-Usmar V.M. (2012). Integration of Cellular Bioenergetics with Mitochondrial Quality Control and Autophagy. Biol. Chem..

[63] Marin D.E., Taranu I. (2012). Overview on Aflatoxins and Oxidative Stress. Toxin Rev..

[64] Zorova L.D., Popkov V.A., Plotnikov E.Y., Silachev D.N., Pevzner I.B., Jankauskas S.S., Babenko V.A., Zorov S.D., Balakireva A.V., Juhaszova M. (2018). Mitochondrial Membrane Potential. Anal. Biochem..

[65] Bayir H. (2005). Reactive Oxygen Species. Crit. Care Med..

[66] Feissner R.F., Skalska J., Gaum W.E., Sheu S.S. (2009). Crosstalk Signaling between Mitochondrial Ca2+ and ROS. Front. Biosci..

[67] Sheik Abdul N., Marnewick J.L. (2020). Fumonisin B1-Induced Mitochondrial Toxicity and Hepatoprotective Potential of Rooibos: An Update. J. Appl. Toxicol..

[68] Nicholls D.G. (2004). Mitochondrial Membrane Potential and Aging. Aging Cell.

[69] Ly J.D., Grubb D.R., Lawen A. (2003). The Mitochondrial Membrane Potential (Δψm) in Apoptosis; an Update. Apoptosis.

[70] Brookes P.S., Yoon Y., Robotham J.L., Anders M.W., Sheu S.S. (2004). Calcium, ATP, and ROS: A Mitochondrial Love-Hate Triangle. Am. J. Physiol. Cell Physiol..

[71] Twarużek M., Zastempowska E., Soszczyńska E., Ałtyn I. (2018). The Use of in Vitro Assays for the Assessment of Cytotoxicity on the Example of MTT Test. Folia Biol. Oecol..

[72] Orellana E., Kasinski A. (2016). Sulforhodamine B (SRB) Assay in Cell Culture to Investigate Cell Proliferation. Bio-Protocol.

[73] Vissenaekens H., Grootaert C., Rajkovic A., Van De Wiele T., Calatayud M. (2019). The Response of Five Intestinal Cell Lines to Anoxic Conditions in Vitro. Biol. Cell.

